# Sodium Alginate Decreases the Concentration of Calcium in Wines, Possibly Lowering the Risk of Calcium Tartrate Instability

**DOI:** 10.3390/foods15081354

**Published:** 2026-04-13

**Authors:** V. Felipe Laurie, Bárbara Hormazabal-Moya, Ricardo I. Castro, Cristina Ubeda, Mariona Gil i Cortiella

**Affiliations:** 1 Laboratorio de Química Enológica, Departamento de Horticultura, Facultad de Ciencias Agrarias, Universidad de Talca, Talca 3460000, Chile; barbara14moya@gmail.com; 2 Centro de Investigación e Innovación VitiScience-CIA250013, Santiago 7820436, Chile; 3 Multidisciplinary Agroindustry Research Laboratory, Universidad Autónoma de Chile, Talca 3467987, Chile; ricardo.castro@uautonoma.cl; 4 Departamento de Nutrición y Bromatología, Toxicología y Medicina Legal, Facultad de Farmacia, Universidad de Sevilla, 41012 Sevilla, Spain; c_ubeda@us.es; 5 Departamento de Bioquímica y Biotecnología, Facultat d’Enologia, Universitat Rovira i Virgili, 43003 Tarragona, Spain; mariona.gil@urv.cat

**Keywords:** calcium, tartrate, stability, sodium alginate, colloid, crystal, wine, dealcoholized, chelation

## Abstract

Calcium tartrate (CaT) instability in bottled wines has become a recurrent issue. Conventional stabilization strategies, such as cold stabilization or the use of protective colloids, can be ineffective or yield inconsistent results. Cation-exchange resins and electrodialysis can reduce the risk of CaT precipitation, but their use is limited by cost and availability. Therefore, the aim of this study was to assess sodium alginate, a natural divalent metal chelator, as a processing aid to remove calcium and improve CaT stability. The study began with the characterization of the alginate composites formed in wines, followed by an evaluation of different doses and contact times. Subsequently, a series of conventional and dealcoholized wines was evaluated, showing significant reductions in calcium concentration (i.e., 27–32% in conventional wines and 10–21% in dealcoholized or reduced-alcohol wines) and improved CaT stability. Besides a significant increase in sodium content, conductivity, and turbidity (*p* < 0.05), most other compositional parameters remained stable, with variations observed only in some wines for certain parameters (e.g., CIELab parameters). These findings demonstrate the potential of sodium alginate as a practical calcium-binding agent and suggest the need for further studies to continue evaluating its applicability in winemaking.

## 1. Introduction

Wine quality depends on a combination of physicochemical, microbiological, and sensorial factors. Among these, the physical stability of bottled wines is particularly important, as the appearance of precipitates constitutes a visual defect that can negatively affect consumer perception [1,2].

Recent publications suggest that calcium tartrate (CaT) instability in wines is no longer an occasional phenomenon but has become more frequent [3,4,5]. This trend has been associated with grape compositional changes linked to climate change, including higher pH values [6], which are known to favor CaT formation. Moreover, heat and water deficit may increase calcium concentrations in grapes, as a response to stress, potentially leading to higher calcium levels in wines [7]. Furthermore, certain viticultural (e.g., liming with calcium carbonate) and winemaking practices (e.g., wine fining with calcium bentonite or the use of uncoated cement tanks) may also contribute to a higher incidence of CaT instability [8,9,10].

Calcium tartrate forms through the interaction of calcium (Ca^2+^) and tartrate (T^2−^) ions, particularly at higher pH, where a larger proportion of tartaric acid exists in its dianionic form. Similarly to potassium bitartrate (KHT), a supersaturated state of soluble CaT species can develop, eventually leading to the growth and precipitation of insoluble rhomboid crystals, whose presence in bottled wines can result in consumer rejection [1,2,11,12]. The occurrence of such residues represents a serious problem, given that its formation is difficult to anticipate and it typically develops slowly after bottling [1,2]. Unlike KHT, the nucleation of CaT is not effectively triggered by low temperatures, making cold stabilization strategies largely ineffective [1,2]. Similarly, the use of protective colloids, which are normally successful in preventing KHT deposits (i.e., carboxymethylcellulose and potassium polyaspartate), has shown inconsistent results, improving CaT stability in only some of the wines tested [3,4].

The predisposing conditions for CaT formation include high concentrations of Ca^2+^ and T^2−^ (favored at high pH), low concentrations of potential inhibitors of nucleation or crystal growth (i.e., malic, citric, or uronic acids), and elevated ethanol concentrations [12,13,14,15,16]. Calcium content in wines ranges from 7 to 310 mg/L, varying widely depending on the grape origin and viticultural and winemaking practices [2,17,18,19]. In contrast, tartaric acid is typically present at concentrations between 2 and 6 g/L and is mainly influenced by grape variety, ripeness, climate, and winemaking practices [1,2].

As pH increases, the equilibrium of tartaric acid favors its dianionic form (T^2−^) which is responsible for complexing with calcium [2]. Malolactic fermentation is of particular relevance as it increases wine pH and replaces malic acid with lactic acid, thus reducing one of the potential inhibitors of calcium salt formation. Similarly, higher ethanol concentrations decrease CaT solubility [14], making nucleation and crystal growth more favorable. Conversely, high-molecular-weight compounds (i.e., proteins and tannins) may bind calcium or tartrate ions, potentially limiting CaT formation [4].

Ribéreau-Gayon et al. (1997) noted that bottled wines above 60 mg/L in reds and 80 mg/L in whites are at risk of becoming unstable [1]. Although this information has been widely reproduced, these limits should be followed with caution, as calcium concentration is just one of several predisposing factors explaining this phenomenon.

Industrial alternatives to address this issue include electrodialysis, which can effectively remove large amounts of cations such as Ca^2+^ [2,20,21]. Additionally, cation exchange resins, which are primarily intended for pH adjustment, can also reduce calcium [22,23], though less effectively than electrodialysis. Both methods may help reduce the risk of CaT instability by reducing the concentration of Ca^2+^ in wine. In contrast to ion-removal techniques, an alternative approach involves promoting the precipitation of CaT itself. For instance, seeding wines with micronized CaT, along with agitation, has been shown to favor crystal growth and precipitation of CaT crystals. However, these results may vary depending on the purity of the CaT crystals employed [13]. The addition of D- or DL-tartaric acid to form calcium DL-tartrate is considered risky and not recommended, as precipitation could continue after bottling [24].

More recently, calcined zeolites showed significant reductions in Ca^2+^, but this was only tested in one white wine [25]. In contrast, carrageenan [5] and alginic acid [4], both of which are natural polysaccharides extracted from seaweeds, have been tested in a variety of wines, showing significant improvements in CaT stability.

Alginates are naturally occurring polysaccharides from algae and some bacteria, consisting of sequences of mannuronic and guluronic acids, which have attracted significant attention due to their multiple uses in the biomedical, food, and agricultural industries [26,27,28]. Their solubility in water-based solutions depends on the pH, ionic strength, and the availability of gelling ions, such as divalent cations, including Ca^2+^ [26]. Although present at concentrations 100–1000 times lower than Ca^2+^, other divalent cations such as Cu^2+^, Zn^2+^ and Mn^2+^ may also potentially be chelated by alginates. In water-based systems, these interactions have been shown to occur with different affinities and gelling mechanisms [29,30]. Sodium alginate is a non-hazardous and biodegradable polysaccharide, which has been regarded as environmentally friendly and is commonly used in food and other applications.

Therefore, the study addresses the persistent problem of CaT instability in bottled wines. Sodium alginate is proposed as a natural ion-binding polymer capable of partially removing calcium ions from wines, thereby reducing CaT formation. Although sodium alginate has been previously studied in winemaking for the immobilization of yeast, bacteria, or enzymes [31,32,33], its potential to reduce calcium concentration and limit CaT precipitation has not been systematically evaluated. The objective of this study was to assess the effectiveness of sodium alginate in reducing calcium concentration in wines, thereby limiting the risk of calcium tartrate instability, and to evaluate other chemical changes resulting from this treatment.

## 2. Materials and Methods

The experimental approach involved an assessment of the gels formed after adding the alginate salt, followed by a trial testing different doses and contact times. The best conditions tested were then applied to different wines to assess compositional changes and potential improvements in calcium tartrate stability, as follows:

### 2.1. Assessment of Alginate Gel Formation

A bottled Sauvignon Blanc (2024 vintage), from Maule, Chile, containing 37.81 mg/L of calcium was treated with 2 g/L of sodium alginate (alginic acid sodium salt from brown algae, CAS No 9005-38-3, Sigma-Aldrich, Darmstadt, Germany) and lightly agitated for 0, 2, and 4 h (Allsheng OS-200 orbital shaker, Hangzhou, China). Then, the samples were centrifuged at ~12,000× *g* for 5 min, and their residues were recovered to verify the formation of gelled structures. To improve visualization under the microscope, the samples were stained with acid fuchsin (Sigma-Aldrich) and photographed at 40× magnification using a stereomicroscope (Olympus SZ-61TR, Tokyo, Japan).

Thermogravimetric analyses (TGA) of the alginate residues were performed using an STD 650 thermal analyzer (TA instruments, New Castle, DE, USA), with 10 mg of each sample placed into Pt crucibles. The temperature program ranged from 50 to 600 °C at a heating rate of 10 °C/min. High-purity nitrogen (99.999%, Indura, Santiago, Chile) was used as the protective gas for mass analyses, at a flow rate of 50 mL/min.

### 2.2. Evaluation of the Alginate Dosage and Contact Time

A trial was conducted to evaluate the removal of calcium after different dosages and contact times of sodium alginate in white and red wine. Four doses (0, 300, 600, and 1200 mg/L) and four contact times (0, 4, 8, and 16 h) were tested in triplicate, using commercial Sauvignon Blanc (vintage 2025; 84.35 mg/L Ca) or Cabernet Sauvignon wines (vintage 2024; 75.53 mg/L Ca). Treatments were carried out by adding sodium alginate at different doses to 100 mL wine samples in amber glass bottles. The bottles were placed horizontally on an orbital shaker (Allsheng OS-200, Hangzhou, China) and kept under constant agitation for the designated times. Once each contact time was completed, the samples were centrifuged at 19,000× *g* for 15 min at 20 °C (TG1650-WS-Fascio, HES, Santiago, Chile), and the resulting supernatants were filtered (0.45 μm, PTFE syringe filters, Jet Biofil, Guangzhou, China) prior to analysis.

Calcium content before and after each treatment was measured using a commercial colorimetric kit based on Arzenazo III (Code 12824, Biosystems, Barcelona, Spain), processed with an automatic wine analyzer (Y15 from Biosystems). Additionally, free ionized calcium was measured with an ion-selective electrode (ISE-HI4104 from Hanna Instruments, Woonsocket, RI, USA), connected to a benchtop pH/ISE/EC meter (HI5522-02, Hanna Instruments). The use of these simple and complementary methods provided a quick and more comprehensive assessment of the effectiveness of sodium alginate in reducing calcium.

### 2.3. Wine Compositional Changes After Sodium Alginate Treatments

Six wine samples with moderately high calcium concentrations (67.7 to 96.3 mg/L of Ca), previously cold-stabilized for KHT (−4 °C for two weeks), were used in this trial. Experiments were conducted in triplicate with 100 mL of sample in amber bottles, comparing untreated controls with samples treated with sodium alginate. Based on the preliminary tests described in Section 2.2, a sodium alginate dose of 1200 mg/L and a contact time of 8 h (h) were selected for these trials.

For each wine, six bottles of 100 mL each (three controls and three alginate-treated samples) were placed on an orbital shaker and continuously agitated for 8 h at room temperature. Then, the samples were centrifuged (19,000× *g*, 15 min, 20 °C), filtered through 0.45 µm PTFE syringe filters, and analyzed as described below (Section 2.3.1 and Section 2.3.2).

These experiments were repeated using a second orbital shaker to obtain additional samples, which were frozen in 50 mL centrifuge tubes for gas chromatographic or backup analyses (−40 °C). Before analysis, these samples were thawed at 5 °C, equilibrated to 20 °C, filtered (0.45 µm), and analyzed as described in Section 2.3.3.

#### 2.3.1. General Physicochemical Measurements

Wine pH and conductivity were measured with a benchtop pH/ISE/EC meter, model HI5522-02 (Hanna Instruments), and ethanol (% *v*/*v*) was estimated by ebulliometry (Dujardin-Salleron^®^, Noizay, France). Turbidity was measured with an HI 83749 turbidimeter (Hanna Instruments) after calibration with AMCO-AEPA-1 standards (Hanna Instruments). The sum of glucose and fructose (G-F), total acidity, tartaric acid, total polyphenols, anthocyanins, catechins, free and total sulfur dioxide (SO_2_), calcium (Ca), and iron (Fe) were measured with an automatic wine analyzer based on UV-Vis spectroscopy (Y15, Biosystems), employing chemical or enzymatic colorimetric kits (codes 12819, 12846, 12808, 12815, 12831, 12834, 12813, 12806, 12824, and 12817) from Biosystems.

The total content of Ca, potassium (K), and sodium (Na) was determined by atomic absorption spectroscopy (AAS). In brief, samples were digested in a microwave oven (MarsXpress, CEM Corporation, Matthews, NC, USA) and analyzed using a flame AAS (FAAS 280, from Agilent, Santa Clara, CA, USA) operated under standard conditions with an air-acetylene flame, including blanks for quality control. The wavelengths used were 422.7 nm for Ca, 766.5 nm for K, and 589.0 nm for Na. Quantification was based on calibration curves prepared from reference standard solutions (Certipur^®^, Merck, Darmstadt, Germany), with coefficients of determination (R^2^) above 0.995.

Wine color was assessed using CIELab space coordinates (L, a*, b*) calculated with MSCV^®^ software (Grupo de Color, Departamento de Química, Universidad de la Rioja, Logroño, Spain), based on absorbance readings at 450, 520, 570, and 630 nm. Measurements were performed with a microplate spectrophotometer (EpochTM Biotek, Agilent, Santa Clara, CA, USA), using 96-well flat bottom polystyrene plates (350 μL of capacity; Brand, Wertheim, Germany), as proposed by Pérez-Caballero et al. (2003) [34]. In addition, absorbance at 420 nm was recorded for white wines to assess browning.

Total phenolics were measured with the Folin–Ciocalteu micro-volume assay [35], using Folin–Ciocalteu reagent (2 N) and sodium carbonate (≥99.5%) from Sigma-Aldrich, and a Synergy HTX multi-mode plate reader (BioTek Instruments, now part of Agilent technologies, Santa Clara, CA, USA). For the total phenolic index (TPI), diluted wine samples (i.e., 1:10 for white wines and 1:100 for reds) were measured at 280 nm in UV-transparent 96-well microplates (Brand, Wertheim, Germany).

Condensed tannins were evaluated in red wine samples using the methylcellulose precipitable (MCP) method, in the 1 mL format, as described by Mercurio et al. (2007) [36], using methylcellulose (1500 cP) and ammonium sulfate (99%) from Sigma-Aldrich. In this case, the same microplates and spectrophotometer indicated for wine color analysis were used.

#### 2.3.2. Soluble Polysaccharide Profiles

The content of soluble polysaccharides before and after the alginate treatments was estimated using high-resolution size-exclusion chromatography, with a refractive index detector (HRSEC-RID) as previously reported elsewhere [3,37]. Wine samples (10 mL) were concentrated to 2 mL with a centrifugal vacuum concentrator (CentriVap, Labconco, Merck), mixed with 10 mL of cold acidified ethanol (0.3 M HCl), and stored at 4 °C for 24 h to promote polysaccharide precipitation. Then, samples were centrifuged (20,000× *g*, 10 min, 4 °C), and the pellet formed was recovered, washed (rinsed twice with cold absolute ethanol), and redissolved in ultrapure water (1 mL), transferred to centrifuge tubes, stored at −80 °C, and lyophilized (FreeZone Legacy 2.5 L, Labconco, Merck). The dry extracts were then dissolved in 1 mL of 30 mM ammonium formate, filtered (0.45 µm pore size, Millex^®^–GV, Merck-Millipore), and injected (100 µL) into the chromatograph (Agilent 1260, with a refractive index detector. Agilent Technologies). Separation was carried out under isocratic conditions (0.6 mL/min) using 30 mM ammonium formate as the mobile phase and two Shodex OHpak SB-803 HQ and SB-804 HQ(Resonac Corporation, Tokio, Japan) columns connected in series as the stationary phase (20 °C oven temperature).

Column calibration employed a series of dextran standards from *Leuconostoc mesenteroides*, using number-average molecular mass (Mn, kDa). Quantification of polysaccharides was achieved using external standards of dextran (410 kDa) and citrus pectin (20–34% esterified), both from Sigma-Aldrich.

#### 2.3.3. Analysis of Volatile Compounds

The headspace solid-phase microextraction (HS-SPME) of volatiles was performed with a 2 cm Carboxen/DVB/PDMS 50/30 µm SPME fiber (Supelco, Bellefonte, PA, USA). For this, 7.5 mL of each wine replicate was transferred into 20 mL glass vial with 1.5 g of sodium chloride and 4-methyl-2-pentanol as an internal standard. Sample incubation was performed at 40 °C with agitation (250 rpm) for 5 min. After this, the SPME fiber was exposed to the vial’s headspace for 35 min. Sample desorption in splitless mode was performed at 250 °C for 180 s. The chromatograph employed was an Agilent 8890 coupled to an Agilent 5977B simple quadrupole mass spectrometer (Agilent Technologies), combined with an MPS autosampler (Gerstel, Müllheim an der Ruhr, Germany), using a J&W CPWax-57CB column of 50 m × 0.25 mm and 0.25 μm film thickness (Agilent Technologies) and a helium flow rate of 1 mL/min. The oven temperature was programmed with the following ramps: 35 °C for 1 min, increased to 220 °C at 2.5 °C/min and held for 7 min. Compound detection was carried out in full scan mode at 70 eV in the range of 29 to 300 *m*/*z*.

Compound identification was performed using the NIST Mass Spectral database (Version 2.0) and comparing with the linear retention index (LRI) values from the literature, obtained from the retention times of n-alkanes (C10–C40) under identical conditions. The volatile compound data are shown as relative areas with respect to the internal standard.

### 2.4. Evaluation of Calcium Tartrate Stability

The stability of 10 wine samples, before and after the sodium alginate treatments (as indicated in Section 2.3), was assessed using the method proposed by Abguéguen and Boulton [13], with slight modifications. Each condition was evaluated in triplicate (i.e., three bottles per condition per wine).

After the alginate treatments, calcium concentration was measured in both control and alginate-treated samples using the Arzenazo III method (denoted as [Cai]).

To promote calcium tartrate crystallization, each sample received a dose of 4000 mg/L of micronized CaT crystals (Enocristal Ca, Enartis, Trecate, Italy), and a 3 cm magnetic stir bar was added to each bottle. The bottles were then placed on a digital 15-position magnetic stirrer (MultiStirrer 6, Velp, Usmate, Italy) set to gentle agitation for 2 h, after which they were transferred to a cold chamber and kept at 0 °C for 48 h, except for the dealcoholized wine which was sampled after 24 h.

After that time, samples were filtered (0.45 μm) and analyzed to determine the final calcium concentration (denoted as [Caf]). Changes in calcium concentration (ΔCa) were recorded, and the following instability thresholds were used: “unstable” > 25 mg/L, “slightly unstable” 15–25 mg/L, and “stable” < 15 mg/L ΔCa.

### 2.5. Statistical Analyses

Data normality and homoscedasticity were checked using the Shapiro–Wilk and Levene tests, respectively. Factorial analysis of variance (ANOVA) was applied to assess the effects of alginate dose and contact time. Significant ANOVA results (*p* < 0.05) were followed by Tukey’s post hoc test. In contrast, chemical changes between control and alginate-treated wines were compared using Welch’s *t*-test. All analyses were conducted using the software R, version 4.5.2, and graphs were made with GraphPad Prism version 10.6.1 (GraphPad Software, Boston, MA, USA). The graphical abstract was built using Chemix Draw (https://www.chemix.org) and PowerPoint (Microsoft Corp., Redmond, WA, USA).

## 3. Results and Discussion

### 3.1. Assessment of Alginate Gel Formation

**Figure 1:**
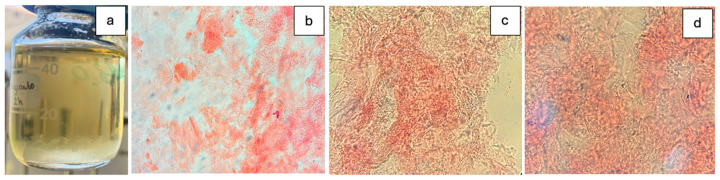
Residues formed after sodium alginate treatment at 2 g/L in white wine under constant agitation. (**a**) White wine with precipitated alginate gel. (**b**–**d**) Microscopy images of alginate gels stained with acid fuchsin (40× magnification) after 0, 2, and 4 h of agitation, respectively.

The addition of sodium alginate to white wine resulted in the formation of aggregates (Figure 1a), which developed greater structural cohesion with increasing contact time, as represented by Figure 1b–d.

**Figure 2:**
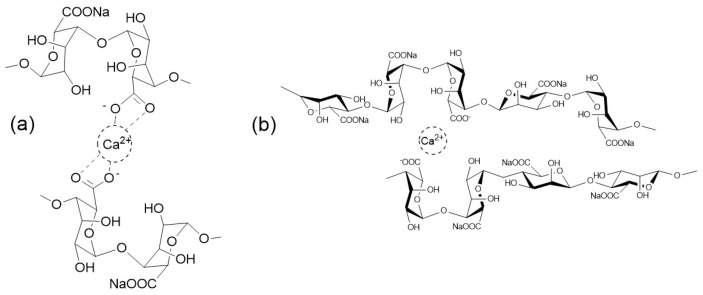
Schematic representation of (**a**) the binding of two alginate chains with a calcium ion, and (**b**) the formation of an “egg-box” type structure.

Most likely, the formation of these composites is due to the coordination between Ca ions and the carboxylate groups of two adjacent alginate chains (Figure 2a). In this case, longer contact times favored the cross-linking of further chains and the formation of thicker calcium alginate gels. This process has been described as the “egg-box” model [38], because the calcium ions occupy the complementary spaces between two alginate chains (Figure 2b), thus allowing for the ionic coordination that keeps the chains together [30].

**Figure 3:**
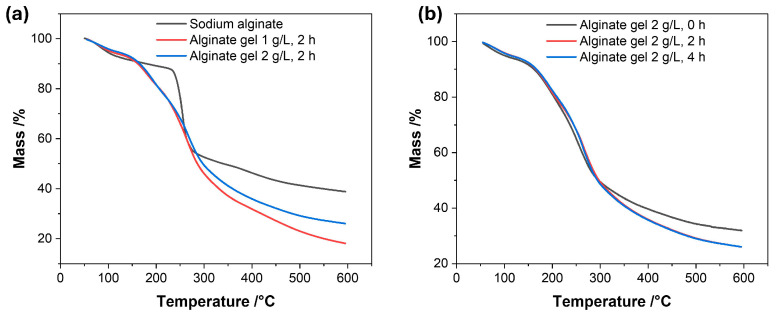
Thermogravimetric curves of (**a**) commercial sodium alginate and the composites recovered from white wine treated with 1 or 2 g/L of sodium alginate (contact time: 2 h), and (**b**) white wine treated with 2 g/L of sodium alginate at different contact times.

The thermogravimetric analysis (TGA) of the recovered alginate composites showed mass-loss patterns that differed from those of commercial sodium alginate (Figure 3a). Sodium alginate exhibited greater thermal stability in the 150–240 °C range than the composites recovered from wine, which is consistent with partial conversion of the polymer into calcium alginate. Between 240 and 280 °C, a rapid mass loss event was observed for the commercial sodium alginate, commonly associated with depolymerization and decarboxylation processes, leading to the formation of sodium carbonate and carbonaceous residues [39].

In contrast, the composites recovered from wine exhibited a broader and less pronounced mass-loss profile in the 150–250 °C range, suggesting the formation of structurally modified materials compared to the original sodium alginate. These changes are consistent with the stabilization of alginate chains through interactions with calcium ions, primarily via ionic coordination, and potentially supported by secondary hydrogen bonding and hydrophobic interactions within the gel network [40].

Additionally, a consistent increase in residual mass was observed with increasing alginate concentration, from 1 g/L to 2 g/L, suggesting the formation of more thermally stable composites at higher polymer loadings. Variations associated with wine contact time suggest that the interaction between wine constituents and the alginate matrix could reach a saturation state, where diffusion and binding within the gel are nearly completed (Figure 3a,b). This may result in composites with stronger intermolecular interactions and enhanced stability.

Considering all the results, these findings indicate that processing conditions influence the structure and stability of alginate residues, which may have important implications for clarification efficiency and residue removal during winemaking.

### 3.2. Evaluation of the Alginate Dosage and Contact Time

**Figure 4:**
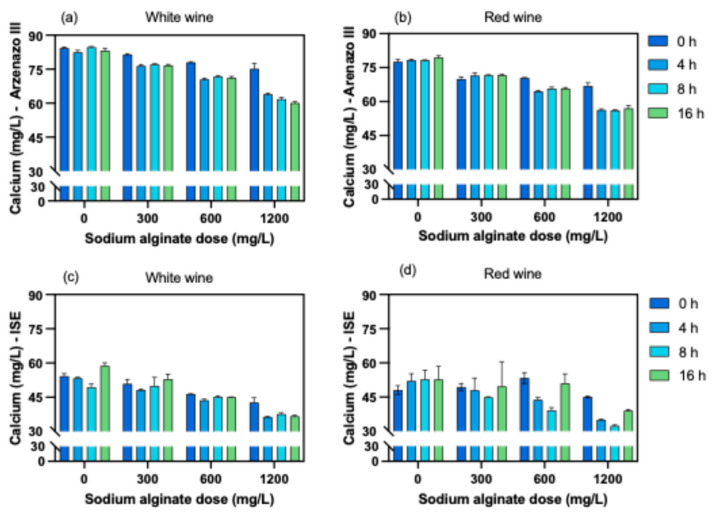
Effect of sodium alginate dose and contact time on calcium concentration in white (**a**,**c**) and red (**b**,**d**) wines, measured by Arzenazo III (**a**,**b**) and ion-selective electrode, ISE (**c**,**d**).

In both white and red wine samples with no alginate addition, calcium concentrations measured with the Arzenazo III method remained relatively stable over the 16 h period (~82–85 and ~77–79 mg/L, respectively). However, increasing the sodium alginate dosage resulted in progressive reductions in calcium concentration. This effect was more pronounced with longer contact times, reaching approximately 60 mg/L in white wine and 57 mg/L in red wine after 16 h of treatment with 1200 mg/L of alginate (Figure 4a,b).

The two-way ANOVA revealed that both factors (i.e., sodium alginate dose and contact time), as well as their interaction, were highly significant (*p* < 0.0001) for calcium reduction. Among these factors, alginate dose was the main contributor, accounting for approximately 78% of the total variation in white wine and 87% in red wine. The significant interaction observed between alginate dose and contact time denotes the interdependence of both factors on calcium removal, although it explained a smaller proportion of the total variation (~7% in the white wine, and ~8% in the red wine sample).

When calcium concentration was assessed using the ion-selective electrode (ISE), greater variability was observed among replicates and contact times, compared to the Arzenazo III measurements. While the general trend showed decreasing calcium concentration with increasing alginate dose and contact time, a slight increase was observed in the red wine treated with 1200 mg/L of alginate at 16 h (Figure 4 c,d). This observation could reflect changes in calcium binding dynamics over extended contact periods, potentially involving slow re-equilibration of calcium ions between bound and free forms or partial depolymerization of the alginate gels [12,41]. However, the contribution of analytical variability to this observation cannot be ruled out. To better elucidate these dynamics, future studies may require direct analysis of the gel deposits.

As observed previously, the two-way ANOVA of the ISE results showed that alginate dose, contact time, and their interaction had significant effects on calcium reduction.

The observed reduction in calcium concentration with increasing alginate dosage and contact time is consistent with the binding of divalent ions through alginate complexation [26,29]. The addition of more alginate may provide additional binding sites for calcium, thereby increasing its removal. In contrast, contact time possibly plays a secondary role by facilitating diffusion and structural rearrangement within the gels formed [30,38].

Based on these results, and the amounts of gel formed, a dose of 1200 mg/L and a contact time of 8 h of were selected for the subsequent experiments evaluating general composition and wine stability.

### 3.3. Wine Compositional Changes After Sodium Alginate Treatments

#### 3.3.1. General Physicochemical Measurements

The treatments with sodium alginate (1200 mg/L, 8 h of contact time) did not produce variations in ethanol, residual sugars, or sulfites, except for a slight decrease in total SO_2_ in the dealcoholized white wine analyzed, most likely due to analytical variability (Table 1). Three of the wines tested showed minor but significant pH increases, ranging from 0.05 to 0.07 units (Table 1), possibly explained by small shifts in the dissociation equilibria of tartaric acid when a significant percentage of calcium is removed from solution. Nevertheless, only one of these wines showed a slight reduction in total acidity (reduced alcohol white wine), and none of them showed differences in tartaric acid. Likewise, no variations in total phenolics, anthocyanins or precipitable tannins (measured only in red wines) were detected.

Colorimetric responses were wine-type-dependent. In white wines, the L* value tended to decrease and b* increased in some of the samples, indicating a slight darkening or yellowing, which was also noted as increases in light absorption at 420 nm. In contrast, one of the red wines tested exhibited higher L*, a*, and b* values, suggesting a lighter color, with a small shift toward yellow/orange tones.

As opposed to the above, significant reductions in calcium concentration across all wine types evaluated, ranging from 10 to 32%, were detected (Table 1). These reductions were noted regardless of the analytical methodology employed, showing good agreement among results (Figure S1). The alginate treatments were less effective in removing calcium from the dealcoholized (10% reduction) and low-alcohol (22% reduction) samples, compared to the conventional wines tested (28 to 32% reduction), possibly because the lack of ethanol increases the solution’s dielectric constant, resulting in weaker crosslinking between alginate and calcium. No statistical differences in potassium were detected for any of the wines tested, and only a minimal but significant reduction in iron content was observed in the dealcoholized wine. In contrast, an important collateral effect of the addition of sodium alginate was observed regarding sodium concentration, which increased by up to ten-fold (but without surpassing 150 mg/L of total Na). It is expected that larger dosages and contact times of sodium alginate would result in even more sodium release, and this option would not be recommended. Related to this, four of the wines tested showed significant increases in conductivity, most likely driven by the rise in sodium concentration (Table 1).

Comprehensive surveys of Californian and Australian wines found that sodium contents fall typically below 100 mg/L, with only a few wines surpassing 200 mg/L [42,43]. Other studies reported concentrations below 70 mg/L for Spanish, Chilean, and Italian wines [18,44,45,46]. Although most countries do not have common regulations for sodium content in wine, the International Organization of Vine and Wine, OIV, suggests a maximum acceptable limit of 80 mg/L of “sodium in excess” [47], which is defined as the content of sodium ions minus the content of chloride ions expressed as sodium [48]. By using the concept of “sodium in excess” rather than total sodium, one could distinguish natural sodium from that eventually introduced during winemaking.

The salty taste perception in wines is a complicated, multifactorial phenomenon, where not only sodium but also chloride ions are required for the activation of salt receptors [49,50]. All in all, potential treatments with sodium alginate should be further studied before considering their commercial use in wines, and if eventually approved, their application should be restricted to wines with low sodium content.

Additionally, the treatments with sodium alginate produced marginal, but significant, increases in wine turbidity in four of the wines tested and important increments in the other two wines (Sauvignon Blanc and Chardonnay) tested (Table 1). These effects might be explained by an incomplete removal of the colloids formed or by the destabilization of the gels formed prior to racking. This effect should be further studied in order to minimize turbidity increments.

Future work could evaluate the affinity of alginate for other divalent cations, such as Mn^2+^ and Cu^2+^, which are important for fermentation and oxidation processes, respectively [1].

#### 3.3.2. Soluble Polysaccharide Profile

**Figure 5:**
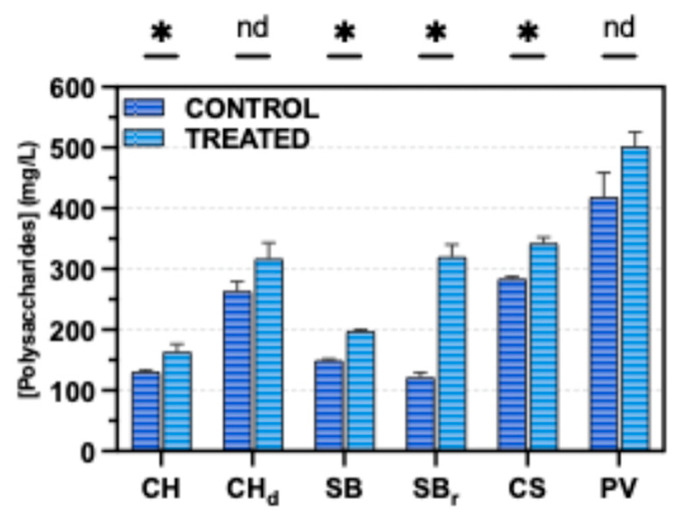
Total soluble polysaccharides of wine samples expressed in mg/L. -* indicatesstatistically significant differences (*p* < 0.05) between control and treated samples based on a *t*-test, while nd denotes no difference. CH: Chardonnay. CHd: Dealcoholized Chardonnay; SB: Sauvignon Blanc; SBr: Reduced-alcohol Sauvignon Blanc; CS: Cabernet Sauvignon; PV: Petit Verdot.

As shown in Figure 5, sodium alginate additions resulted in measurable increases in the content of total soluble polysaccharide in all wine samples. Statistically significant differences between control and treated wines (*p* < 0.05) were observed for Chardonnay (CH), Sauvignon Blanc (SB), reduced-alcohol Sauvignon Blanc (SBr), and Cabernet Sauvignon (CS). Among these, SBr wines showed the most pronounced response, with polysaccharide concentrations increasing by more than twofold relative to the control. Although the dealcoholized Chardonnay (CHd) and Petit Verdot (PV) wines also exhibited higher mean polysaccharide levels following treatment, these increases were not statistically significant, possibly due to greater variability within these samples.

It should be noted that the analysis employed (i.e., HRSEC-RID following ethanol precipitation) provides an estimate of soluble polysaccharides and does not allow for the identification of polysaccharide families or the discrimination between naturally occurring wine polysaccharides and alginate-derived residues. Nevertheless, the consistency of the trends observed across different wine types supports the reliability of the comparative results.

**Figure 6:**
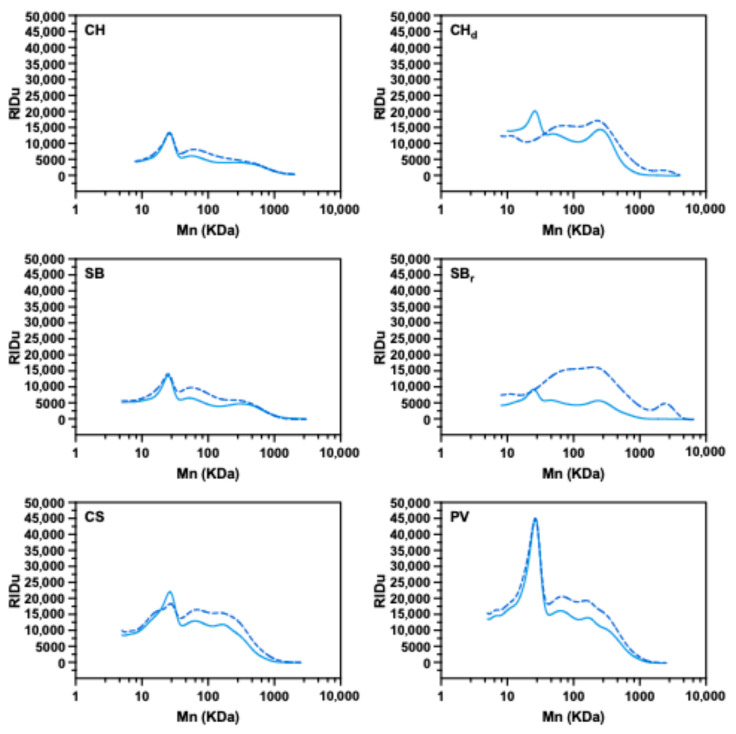
Total Chromatographic profile of soluble polysaccharides of wine samples. Solid light blue line corresponds to control (untreated) wines and dashed blue lines to treated wines. CH: Chardonnay. CHd: Dealcoholized Chardonnay; SB: Sauvignon Blanc; SBr: Reduced-alcohol Sauvignon Blanc; CS: Cabernet Sauvignon; PV: Petit Verdot.

Additionally, Figure 6 shows the average GPC chromatograms of soluble polysaccharides. In conventional (non-dealcoholized) wines, control and alginate-treated samples exhibited similar overall elution patterns, indicating that alginate treatment did not generate new polymeric signals within the range of molecular weights analyzed. However, the alginate treated samples consistently showed higher RI signals between approximately 50 to 1000 kDa. The absence of distinct new peaks suggests that the increased signal cannot be attributed to residual alginate alone. Instead, this may result from changes in the solubility or aggregation state of wine polysaccharides, possibly explained by intermolecular interactions between native polysaccharides and alginate chains or by modifications of the colloidal equilibrium of the wine matrix.

The response of dealcoholized and reduced-alcohol wine differed from that of conventional wines (Figure 6). In both cases, the peak eluting at approximately 50 kDa disappeared after the alginate treatment, suggesting its removal or destabilization to lower-molecular-weight species. From approximately 70 kDa onward, dealcoholized wines showed similar chromatographic patterns than the control but with higher overall signals, suggesting an increase in the solubility of larger-molecular-weight polysaccharides. By contrast, in the reduced alcohol wines, a broader and more intense signal was observed in the range of approximately 60 to 1000 kDa, pointing to a substantial modification of the colloidal matrix.

These differences may be partly explained by the influence of ethanol concentration on polysaccharide solubility and aggregation. Increasing ethanol content has been shown to reduce polysaccharide solubility and promote chain association or precipitation [51]. Accordingly, wines with lower ethanol content may have higher polysaccharide solubility (or altered aggregation states), increasing their sensitivity to alginate addition. Additionally, calcium alginate gels have also shown changes in swelling and structural properties depending on ethanol exposure [52]. Together, these effects may help explain the more pronounced responses observed in reduced-alcohol and dealcoholized wines.

The increase in soluble polysaccharides observed in conventional wines may has important technological and sensory implications, as polysaccharides are known to influence not only mouthfeel but also the inhibition of nucleation and tartrate formation.

#### 3.3.3. Analysis of Volatile Compounds

**Figure 7:**
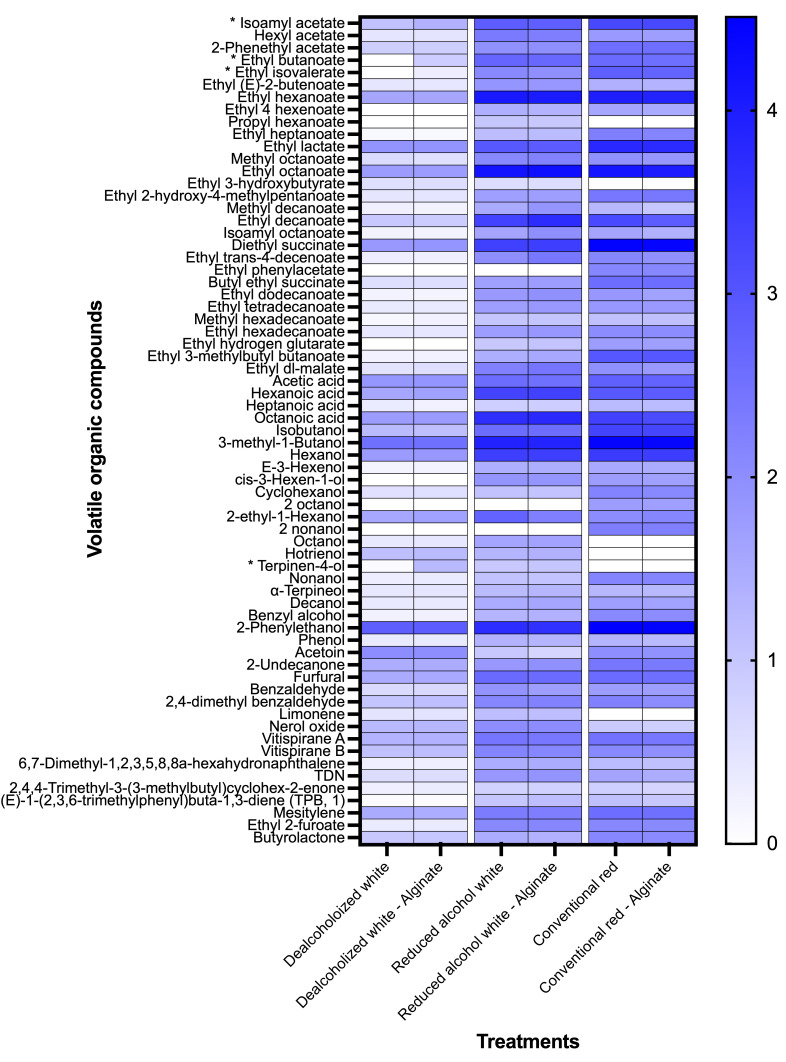
Heatmap of volatile organic compounds detected in untreated and alginate-treated wine samples. (*) Denotes statistically significant differences (*p* < 0.05) between control and treated samples in the dealcoholized wine, according to a *t*-test.

A total of 67 VOCs were identified in the wine samples analyzed, corresponding to esters, carboxylic acids, alcohols, carbonyl compounds, terpenes and terpenoids, norisoprenoids, and other aromatic and cyclic compounds (Figure 7).

In order to perform the *t*-test comparing controls and alginate-treated wines, a logarithmic normalization of the data was required due to the wide range of areas among volatile signals. These analyses show that the effect of the alginate treatment on the volatile profile varied among wine types. The *t*-test showed that statistical differences were only observed in the dealcoholized Chardonnay wine. In this wine, the alginate-treated samples showed a higher amount of isoamyl acetate, ethyl butanoate, ethyl isovalerate, and terpinen-4-ol. The increase likely reflects matrix-dependent interactions during the process. Although alginate was removed before analysis, it may have entrapped more volatiles in ethanol-containing wines, where hydrophobic compounds are better solubilized and bind more strongly to alginate. In dealcoholized wine, lower solubility reduces volatile removal, resulting in higher residual levels. Additionally, salting-out effects in the aqueous dealcoholized matrix may have increased volatile partitioning into the headspace, explaining their elevated detection by HS-SPME-GC-MS [53].

In general, the reduced-alcohol and conventional red wines showed higher overall signal intensities across most volatile compounds compared to the dealcoholized wine, suggesting higher overall volatile concentrations in these wines, which were not significantly affected by alginate treatments (Figure 7).

In future experiments, a more detailed characterization of the volatile profile, along with formal sensory tests, would be advisable to ensure that the small changes observed are not perceived by tasters.

### 3.4. Evaluation of Calcium Tartrate Stability

Table 2 shows the effect of sodium alginate addition on calcium concentration and stability classification for 10 different wines of various types and varieties. The control samples had total calcium concentrations ranging from 55 to 98 mg/L, and their stability classification ranged from stable to unstable, but most of the samples were classified as either unstable or slightly unstable based on the method proposed by Abguéguen and Boulton [13].

The addition of sodium alginate improved the stability classification in most of the wines tested. All samples that were initially classified as slightly unstable or unstable changed to stable after the alginate treatment. This improvement is linked to a reduction in ΔCa, which became close to zero or even negative in some cases. Also, as indicated elsewhere [13], when a notable rise in calcium concentration is observed, the wine is regarded as stable at the test temperature, as this indicates that some dissolution of the CaT seeds has occurred. Nevertheless, negative values may also reflect experimental variability or measurement limitations, so these results should be interpreted as indicative rather than absolute.

The reductions in calcium concentration reported in Table 1, along with the results of the stability test (Table 2), suggest that alginate treatments enhance calcium tartrate stability across different wines. However, the long-term effects of these treatments are yet to be tested.

## 4. Conclusions

The addition of sodium alginate to wine reduced calcium concentration, with decreases ranging from 10 to 21% in dealcoholized and reduced-alcohol wines and 27–32% in conventional wines using 1.2 g/L of sodium alginate with an 8 h contact time. This reduction in calcium helped lower the risk of calcium tartrate instability, showing the potential of sodium alginate as an effective aid for wine stability. Also, the treatments caused significant increases in the sodium level, slight increments in wine turbidity and conductivity, and minor to negligible effects on the other wine chemical variables measured.

These results show that conventional wines respond more strongly to alginate treatment, with higher doses and longer contact times improving calcium removal, but increases in sodium and turbidity indicate that the effects can vary with wine type and conditions.

Further investigation is needed to evaluate long-term stability, potential sensory impacts, and interactions between alginate and other wine components across a broader range of wine styles. Additionally, strategies to minimize sodium and turbidity increases while improving calcium tartrate stabilization should be further examined.

## Figures and Tables

**Table 1:** Effect of sodium alginate treatment (1.2 g/L, 8 h contact time) on selected enological parameters of six wine samples. (*) Denotes statistically significant differences (*p* < 0.05) between control and treated samples according to a *t*-test.

Parameter	Chardonnay-Dealcoholized		Sauvignon Blanc– Low Alcohol		Sauvignon Blanc		Chardonnay		Cabernet Sauvignon		Petit Verdot	
	Control	Na-Alginate	Control	Na-Alginate	Control	Na-Alginate	Control	Na-Alginate	Control	Na-Alginate	Control	Na-Alginate
**Ethanol (% *v*/*v*)**	0.57 ± 0.06	0.50 ± 0.10	9.10 ± 0.10	9.13 ± 0.12	13.9 ± 0.10	13.7 ± 0.32	14.0 ± 0.06	14.2 ± 0.00	13.6 ± 0.12	14.0 ± 0.12 *	15.1 ± 0.10	15.3 ± 0.00
**pH**	3.29 ± 0.00	3.29 ± 0.03	3.17 ± 0.00	3.24 ± 0.00 *	3.09 ± 0.00	3.17 ± 0.00	3.46 ± 0.01	3.53 ± 0.00 *	3.49 ± 0.01	3.54 ± 0.01 *	3.76 ± 0.01	3.77 ± 0.01
**Total acidity (mg/L)**	6.08 ± 0.19	5.96 ± 0.03	5.36 ± 0.09	5.12 ± 0.11 *	5.63 ± 0.06	5.24 ± 0.16	4.10 ± 0.10	4.12 ± 0.29	4.54 ± 0.09	4.49 ± 0.05	4.91 ± 0.02	4.67 ± 0.24
**Tartaric acid (g/L))**	2.66 ± 0.10	2.57 ± 0.03	1.68 ± 0.06	1.78 ± 0.07	2.05 ± 0.01	2.06 ± 0.03	1.50 ± 0.03	1.49 ± 0.03	1.73 ± 0.06	1.72 ± 0.02	1.74 ± 0.03	1.79 ± 0.04
**Glucose/Fructose (g/L)**	20.1 ± 0.29	20.1 ± 0.22	4.25 ± 0.03	4.30 ± 0.04	2.94 ± 0.05	2.90 ± 0.03	5.34 ± 0.11	5.52 ± 0.08	0.99 ± 0.04	1.01 ± 0.02	1.88 ± 0.06	1.96 ± 0.03
**Free sulfites (mg/L)**	34.7 ± 0.58	34.3 ± 0.58	17.0 ± 1.0	17.0 ± 0.00	0.33 ± 0.58	0.67 ± 1.2	7.67 ± 0.58	7.0 ± 0.00	9.00 ± 0.00	9.00 ± 0.00	24.3 ± 1.5	23.0 ± 1.0
**Total sulfites (mg/L)**	137 ± 0.58	136 ± 0.58 *	109 ± 2.1	110 ± 0.58	70.3 ± 2.9	70.3 ± 1.2	82.0 ± 1.7	85.0 ± 1.0	22.7 ± 0.58	24.0 ± 1.73	52.0 ± 1.7	53.0 ± 2.6
**Total phenols (mg GAE/L)**	467 ± 26.2	457 ± 8.9	226 ± 20	197 ± 37	210 ± 33	210 ± 16	311 ± 7.2	313 ± 11	1636 ± 57	1343 ± 145	3867 ± 104	3851 ± 98
**Abs. 420 nm**	0.144 ± 0.00	0.148 ± 0.00 *	0.085 ± 0.00	0.099 ± 0.00 *	0.074 ± 0.00	0.15 ± 0.02 *	0.11 ± 0.00	0.15 ± 0.01	2.61 ± 0.02	2.66 ± 0.07	4.31 ± 0.08	4.31 ± 0.26
**Abs. 520 nm**	-	-	-	-	-	-	-	-	2.80 ± 0.02	2.82 ± 0.08	7.18 ± 0.15	7.08 ± 0.52
L*	98.0 ± 0.00	97.8 ± 0.10	99 ± 0.06	98 ± 0.17 *	98.8 ± 0.15	94.7 ± 1.2 *	98.5 ± 0.06	96.5 ± 0.40 *	24.9 ± 0.72	24.5 ± 0.75	5.83 ± 0.15	6.97 ± 0.12 *
a*	−1.50 ± 0.06	−1.55 ± 0.03	−2.91 ± 0.10	−2.88 ± 0.03	−0.59 ± 0.06	−0.36 ± 0.07	−1.12 ± 0.16	−0.88 ± 0.06	47.3 ± 1.3	47.4 ± 0.22	33.3 ± 0.52	36.1 ± 0.34 *
b*	8.89 ± 0.06	8.93 ± 0.08	5.39 ± 0.06	5.98 ± 0.08 *	4.53 ± 0.11	7.00 ± 0.44 *	6.49 ± 0.07	7.91 ± 0.35 *	39.3 ± 1.2	39.0 ± 0.78	10.0 ± 0.31	12.0 ± 0.22 *
**Anthocyanins (mg/L)**	-	-	-	-	-	-	-	-	108 ± 0.85	106 ± 2.87	621 ± 5.1	621 ± 4.5
**Precipitable tannins (mg/L)**	-	-	-	-	-	-	-	-	1130 ± 106	1213 ± 173	2771 ± 81	2736 ± 99
**Polyphenols (mg/L)**	394 ± 10.6	399 ± 6.4	164 ± 8.1	168 ± 3.7	200 ± 27	199 ± 6.4	264 ± 20	304 ± 22	1295 ± 2.4	1298 ± 13.7	2566 ± 27	2649 ± 13
**Catechins (mg/L)**	137 ± 1.4	140 ± 2.8	28.1 ± 1.03	30.3 ± 6.5	33.9 ± 4.0	34.5 ± 8.0	58.3 ± 3.9	65.8 ± 5.4	383 ± 4.5	384 ± 0.57	>500	>500
**Turbidity (NTU)**	0.65 ± 0.12	1.45 ± 0.19 *	0.63 ± 0.25	2.01 ± 1.4	0.35 ± 0.04	25.8 ± 8.5 *	0.49 ± 0.02	25.3 ± 6.7 *	1.10 ± 0.07	2.88 ± 0.30 *	0.23 ± 0.02	1.24 ± 0.12 *
**Conductivity (µS/cm)**	4083 ± 96.1	4203 ± 153 *	2342 ± 45	2493 ± 5.6 *	1634 ± 33	1767 ± 18 *	1873 ± 25	1958 ± 10.5 *	2458 ± 20	2546 ± 27 *	2682 ± 49	2761 ± 49
**Iron (mg/L)**	4.33 ± 0.02	4.27 ± 0.02 *	1.39 ± 0.03	1.35 ± 0.03	1.45 ± 0.08	1.64 ± 0.44	1.70 ± 0.13	1.51 ± 0.16	1.23 ± 0.08	1.19 ± 0.05	4.00 ± 0.53	4.17 ± 0.18
**Calcium Arzenazo III (mg/L)**	89.9 ± 0.99	80.8 ± 0.74*	96.3 ± 0.83	75.3 ± 1.3 *	85.0 ± 7.5	59.2 ± 5.6*	81.8 ± 2.0	59.2 ± 1.9*	67.7 ± 0.43	46.1 ± 2.2*	77.8 ± 6.8	56.2 ± 4.5 *
**Calcium AAS (mg/L)**	85.7 ± 7.3	78.1 ± 1.6*	111 ± 0.96	80.0 ± 1.6 *	88.4 ± 3.5	63.2 ± 3.6*	84.4 ± 1.5	52.8 ± 0.61*	78.9 ± 0.85	51.7 ± 0.96*	79.3 ± 1.3	52.5 ± 0.69 *
**Calcium ISE (mg/L)**	58.9 ± 3.7	46.2 ± 1.2*	81.6 ± 3.3	53.8 ± 2.2 *	57.1 ± 2.3	38.9 ± 1.4*	50.8 ± 1.0	30.7 ± 1.7*	33.6 ± 1.8	18.7 ± 1.1*	39.4 ± 0.53	24.1 ± 0.85 *
**Potassium AAS (mg/L)**	823 ± 85	872 ± 21	478 ± 19	513 ± 28	310 ± 28	281 ± 51	547 ± 17	524 ± 18	755 ± 20	717 ± 18	1107 ± 21	1095 ± 5.1
**Sodium AAS (mg/L)**	61.4 ± 8.9	148 ± 1.8*	21.9 ± 1.6	113 ± 7.1*	12.6 ± 0.9	113 ± 3.2*	12.9 ± 0.22	114 ± 4.0*	10.8 ± 0.56	114 ± 3.6*	11.0 ± 0.17	116 ± 0.95 *

**Table 2:** Calcium tartrate stability of wine samples treated with sodium alginate addition (1.2 g/L and 8 h of contact time).

		Cai (mg/L)	Caf (mg/L)	ΔCa (mg/L)	Stability
**Dealcoholized wine-Chardonnay**	Control	89.9 ± 0.99	107.6 ± 4.8	−17.7	Stable
	Na-Alginate	80.8 ± 0.74	98.1 ± 3.2	−17.2	Stable
**Reduced alcohol wine-Sauvignon Blanc**	Control	98.7 ± 0.63	79.6 ± 3.4	19.1	Slightly unstable
	Na-Alginate	76.0 ± 0.87	95.0 ± 1.0	−19.0	Stable
**Sauvignon Blanc**	Control	89.2 ± 0.65	79.7 ± 1.5	9.52	Stable
	Na-Alginate	60.6 ± 0.72	81.5 ± 0.8	−20.8	Stable
**Merlot-Rose**	Control	68.9 ± 1.02	37.1 ± 1.3	31.9	Unstable
	Na-Alginate	45.1 ± 1.17	45.4 ± 1.0	−0.26	Stable
**Cabernet Sauvignon-Rose**	Control	57.3 ± 0.59	35.9 ± 3.9	21.4	Slightly unstable
	Na-Alginate	35.2 ± 0.61	43.6 ± 0.9	−8.42	Stable
**Merlot**	Control	93.6 ± 0.16	72.1 ± 1.4	21.5	Slightly unstable
	Na-Alginate	67.6 ± 0.33	63.7 ± 0.6	3.80	Stable
**Cabernet Sauvignon 1**	Control	81.9 ± 0.28	52.8 ± 2.0	29.2	Unstable
	Na-Alginate	55.8 ± 0.40	52.9 ± 1.1	2.91	Stable
**Cabernet Sauvignon 2**	Control	70.4 ± 0.90	44.7 ± 2.5	25.7	Unstable
	Na-Alginate	44.9 ± 1.75	47.1 ± 2.4	−2.24	Stable
**Cabernet Sauvignon 3**	Control	55.4 ± 0.60	43.5 ± 2.3	12.0	Stable
	Na-Alginate	39.6 ± 3.36	41.2 ± 0.9	−1.65	Stable
**Carmenere**	Control	63.8 ± 0.46	36.8 ± 4.9	27.0	Unstable
	Na-Alginate	45.6 ± 1.76	64.2 ± 9.3	−18.6	Stable

## Data Availability

Data will be available upon request.

## Supplementary Materials

The following supporting information can be downloaded at: https://www.mdpi.com/article/10.3390/foods15081354/s1, Figure S1: Linear regressions for calcium concentration measurements performed with (a) Atomic absorption spectroscopy (AAS) and Arzenazo III, (b) AAS and Ion-selective electrode (ISE), and (c) ISE and Arzenazo III.

## Abbreviations

The following abbreviations are used in this manuscript:

CaT	Calcium tartrate
KHT	Potassium tartrate
T^2−^	Tartrate ion
TGA	Thermogravimetric analysis
PTFE	Polytetrafluoroethylene
G-F	Glucose + Fructose
SO_2_	Sulfur dioxide
FAAS	Flame atomic absorption spectroscopy
CIELab	CIE color system (*Commission Internationale de l’Éclairage*)
TPI	Total phenolics index
HRSEC-RID	High resolution, size exclusion chromatography, with a refractive index detector
HCl	Hydrochloric acid
HS-SPME	Headspace solid phase microextraction
NIST	National Institute of Standards and Technology
LRI	Linear retention index
[Ca_i_]	Initial calcium concentration
[Ca_f_]	Final calcium concentration
ANOVA	Analysis of variance
GPC	Gel Permeation Chromatography
RI	Refractive index
VOC	Volatile organic compounds

## References

[1] Ribéreau-Gayon P., Glories Y., Maujean A., Dubourdieu D. (2000). Handbook of Enology, The Chemistry of Wine Stabilization and Treatments.

[2] Waterhouse A.L., Sacks G.L., Jeffery D.W. (2024). Understanding Wine Chemistry.

[3] Cisterna-Castillo M., Covarrubias J.I., Medel-Marabolí M., Peña-Neira A., Cortiella M.G.I. (2024). Influence of Protective Colloids on Calcium Tartrate Stability and the Astringency Perception in a Red Wine. Foods.

[4] Cosme F., Filipe-Ribeiro L., Coixao A., Bezerra M., Nunes F.M. (2024). Efficiency of Alginic Acid, Sodium Carboxymethylcellulose, and Potassium Polyaspartate as Calcium Tartrate Stabilizers in Wines. Foods.

[5] Fioschi G., Prezioso I., Sanarica L., Pagano R., Bettini S., Paradiso V.M. (2024). Carrageenan as possible stabilizer of calcium tartrate in wine. Food Hydrocoll..

[6] van Leeuwen C., Darriet P. (2016). The Impact of Climate Change on Viticulture and Wine Quality. J. Wine Econ..

[7] Keller M. (2020). The Science of Grapevines.

[8] Quiroga M.J., Olego M.A., Sánchez-García M., Medina J.E., Visconti F., Coque J.J.R., Jimeno J.E.G. (2017). Effects of Liming on Soil Properties, Leaf Tissue Cation Composition and Grape Yield in a Moderately Acid Vineyard Soil. Influence on Must and Wine Quality. Oeno One.

[9] Catarino S., Madeira M., Monteiro F., Rocha F., Curvelo-García A.S., de Sousa R.B. (2008). Effect of Bentonite Characteristics on the Elemental Composition of Wine. J. Agric. Food Chem..

[10] Anastasiou E.K., Liapis A., Tsardaka E.C., Chortis A., Gerovassiliou A. (2025). Testing Concrete for the Construction of Winemaking Tanks. Appl. Sci..

[11] McKinnon A.J., Scollary G.R., Solomon D.H., Williams P.J. (1994). The Mechanism of Precipitation of Calcium L-(+)-tartrate in a Model Wine Solution. Colloids Surf. A-Physicochem. Eng. Asp..

[12] McKinnon A.J., Williams P.J., Scollary G.R. (1996). Influence of uronic acids on the spontaneous precipitation of calcium L-(+)-tartrate in a model wine solution. J. Agric. Food Chem..

[13] Abgueguen O., Boulton R.B. (1993). The Crystallization Kinetics of Calcium Tartrate from Model Solutions and Wines. Am. J. Enol. Vitic..

[14] Berg H.W., Keefer R.M. (1959). Analytical Determination of Tartrate Stability in Wine. II. Calcium Tartrate. Am. J. Enol. Vitic..

[15] Mckinnon A.J., Scollary G.R., Solomon D.H., Williams P.J. (1995). The Influence of Wine Components on the Spontaneous Precipitation of Calcium L(+)-Tartrate in a Model Wine Solution. Am. J. Enol. Vitic..

[16] Pellerin P., Doco T., Scollary G.R. (2013). The influence of wine polymers on the spontaneous precipitation of calcium tartrate in a model wine solution. Int. J. Food Sci. Technol..

[17] Hopfer H., Nelson J., Collins T.S., Heymann H., Ebeler S.E. (2015). The combined impact of vineyard origin and processing winery on the elemental profile of red wines. Food Chem..

[18] Laurie V.F., Villagra E., Tapia J., Sarkis J.E.S., Hortellani M.A. (2010). Analysis of major metallic elements in Chilean wines by atomic absorption spectroscopy. Cienc. E Investig. Agrar..

[19] Pohl P. (2007). What do metals tell us about wine?. Trac-Trends Anal. Chem..

[20] Gonçalves F., Fernandes C., dos Santos P.C., de Pinho M.N. (2003). Wine tartaric stabilization by electrodialysis and its assessment by the saturation temperature. J. Food Eng..

[21] Benitez J.G., Macias V.M.P., Gorostiaga P.S., Lopez R.V., Rodriguez L.N. (2003). Comparison of electrodialysis and cold treatment on an industrial scale for tartrate stabilization of sherry wines. J. Food Eng..

[22] Ibeas V., Correia A.C., Jordao A.M. (2015). Wine tartrate stabilization by different levels of cation exchange resin treatments: Impact on chemical composition, phenolic profile and organoleptic properties of red wines. Food Res. Int..

[23] Ponce F., Mirabal-Gallardo Y., Versari A., Laurie V.F. (2018). The use of cation exchange resins in wines: Effects on pH, tartrate stability, and metal content. Cienc. E Investig. Agrar..

[24] (2016). Calcium and Its Unpredictable Presence.

[25] Reilly T., Mierczynski P., Suwanto A., Krido Wahono S., Maniukiewicz W., Vasilev K., Bindon K., Mierczynska-Vasilev A. (2023). Using Zeolites to Cold Stabilize White Wines. Aust. J. Grape Wine Res..

[26] Pawar S.N., Edgar K.J. (2012). Alginate derivatization: A review of chemistry, properties and applications. Biomaterials.

[27] Cazón P., Velazquez G., Ramírez J.A., Vázquez M. (2017). Polysaccharide-based films and coatings for food packaging: A review. Food Hydrocoll..

[28] Beaumont M., Tran R., Vera G., Niedrist D., Rousset A., Pierre R., Shastri V.P., Forget A. (2021). Hydrogel-Forming Algae Polysaccharides: From Seaweed to Biomedical Applications. Biomacromolecules.

[29] Mørch Ý.A., Donati I., Strand B.L., Skjåk-Bræk G. (2006). Effect of Ca^2+^, Ba^2+^, and Sr^2+^ on Alginate Microbeads. Biomacromolecules.

[30] Hu C., Lu W., Mata A., Nishinari K., Fang Y. (2021). Ions-induced gelation of alginate: Mechanisms and applications. Int. J. Biol. Macromol..

[31] Fumi M.D., Trioli G., Colagrande O. (1987). Preliminary Assessment on the Use of Immobilized Yeast-Cells in Sodium Alginate for Sparkling Wine Processes. Biotechnol. Lett..

[32] Shukla S., Park J.H., Kim M. (2020). Efficient, Safe, Renewable, and Industrially Feasible Strategy Employing *Bacillus Subtilis* with Alginate Bead Composite for the Reduction of Ochratoxin A from Wine. J. Clean. Prod..

[33] Fernández-Pacheco P., García-Béjar B., Pérez A.B., Arévalo-Villena M. (2021). Free and Immobilised β-Glucosidases in Oenology: Biotechnological Characterisation and Its Effect on Enhancement of Wine Aroma. Front. Microbiol..

[34] Pérez-Caballero V., Ayala F., Echávarri J.F., Negueruela A.I. (2003). Proposal for a New Standard OIV Method for Determination of Chromatic Characteristics of Wine. Am. J. Enol. Vitic..

[35] Waterhouse A.L. (2005). Determination of Total Phenolics. Handbook of Food Analytical Chemistry.

[36] Mercurio M.D., Dambergs R.G., Herderich M.J., Smith P.A. (2007). High throughput analysis of red wine and grape Phenolics-Adaptation and validation of methyl cellulose precipitable tannin assay and modified Somers color assay to a rapid 96 well plate format. J. Agric. Food Chem..

[37] Fanzone M., Peña-Neira A., Gil M., Jofré V., Assof M., Zamora F. (2012). Impact of phenolic and polysaccharidic composition on commercial value of Argentinean Malbec and Cabernet Sauvignon wines. Food Res. Int..

[38] Grant G.T., Morris E.R., Rees D.A., Smith P.J.C., Thom D. (1973). Biological interactions between polysaccharides and divalent cations: The egg-box model. FEBS Lett..

[39] Zohuriaan M.J., Shokrolahi F. (2004). Thermal studies on natural and modified gums. Polym. Test..

[40] Levic S., Djordjevic V., Rajic N., Milivojevic M., Bugarski B., Nedovic V. (2013). Entrapment of ethyl vanillin in calcium alginate and calcium alginate/poly(vinyl alcohol) beads. Chem. Pap..

[41] Oroná J.D., Zorrilla S.E., Peralta J.M. (2024). Assessment of Calcium Alginate Gels as Wall Materials for Encapsulation Systems. J. Sci. Food Agric..

[42] Berg H.W., Akiyoshi M., Amerine M.A. (1979). Potassium and Sodium Content of California Wines. Am. J. Enol. Vitic..

[43] Martin A.E., Watling R.J., Lee G.S. (2012). The multi-element determination and regional discrimination of Australian wines. Food Chem..

[44] Jurado J.M., Alcázar Á., Palacios-Morillo A., de Pablos F. (2012). Classification of Spanish DO white wines according to their elemental profile by means of support vector machines. Food Chem..

[45] Fermo P., Comite V., Sredojević M., Ćirić I., Gašić U., Mutić J., Baošić R., Tešić Ž. (2021). Elemental Analysis and Phenolic Profiles of Selected Italian Wines. Foods.

[46] Mirabal-Gallardo Y., Caroca-Herrera M.A., Muñoz L., Meneses M., Laurie V.F. (2018). Multi-element analysis and differentiation of Chilean wines using mineral composition and multivariate statistics. Cienc. E Investig. Agrar..

[47] (2025). OIV International Code of Œnological Practices.

[48] (2025). OIV Compendium of International Methods of Wine and Must Analysis.

[49] de Loryn L.C., Petrie P.R., Hasted A.M., Johnson T.E., Collins C., Bastian S.E.P. (2014). Evaluation of Sensory Thresholds and Perception of Sodium Chloride in Grape Juice and Wine. Am. J. Enol. Vitic..

[50] Walker R.R., Holt H., Blackmore D.H., Pearson W., Clingeleffer P.R., Francis L. (2023). Salt concentration and salty taste perception in “Chardonnay” and “Shiraz” wines from own roots and different rootstocks under saline irrigation. Vitis.

[51] Bouchard A., Hofland G.W., Witkamp G.-J. (2007). Properties of Sugar, Polyol, and Polysaccharide Water-Ethanol Solutions. J. Chem. Eng. Data..

[52] Hermansson E., Schuster E., Lindgrenc L., Altskär A., Ström A. (2016). Impact of solvent quality on the network strength and structure of alginate gels. Carbohydr. Polym..

[53] Robinson A.L., Ebeler S.E., Heymann H., Boss P.K., Solomon P.S., Trengove R.D. (2009). Interactions Between Wine Volatile Compounds and Grape and Wine Matrix Components Influence Aroma Compound Headspace Partitioning. J. Agric. Food Chem..

