# Supplementary material for: Sodium Alginate Decreases the Concentration of Calcium in Wines, Possibly Lowering the Risk of Calcium Tartrate Instability
Source: Foods. 2026 Apr 13;15(8):1354. doi: 10.3390/foods15081354 (PMC13114400; doi:10.3390/foods15081354)
Supplement: Supplementary file 1 [file foods-15-01354-s001.zip › foods-4123915-supplementary.pdf]

Supporting information

Sodium alginate decreases the concentration of calcium in wines, possibly lowering the risk of calcium tartrate instability

V. Felipe Laurie*, Bárbara Hormazabal-Moya, Ricardo I. Castro, Cristina Ubeda, and Mariona Gil i Cortiella

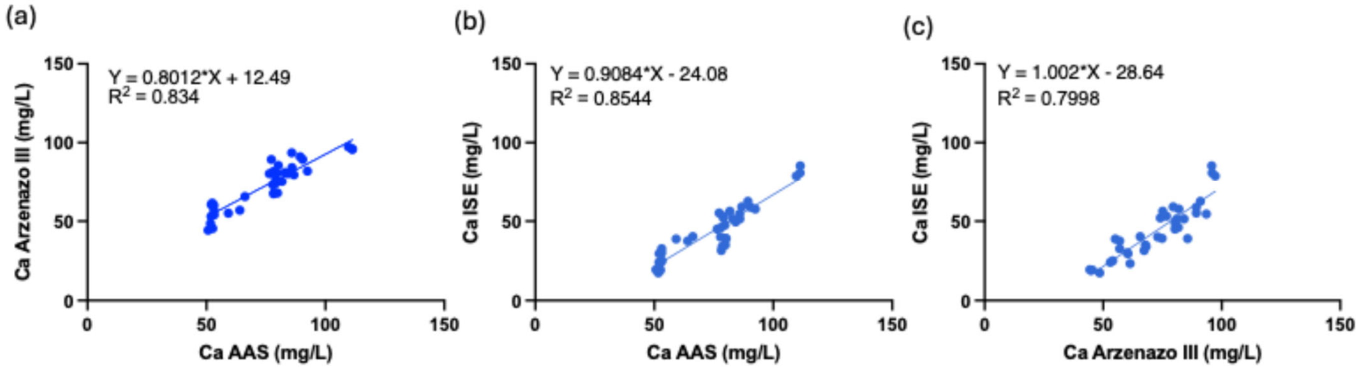

Figure S1. Linear regressions for calcium concentration measurements performed with (a) Atomic absorption spectroscopy (AAS) and Arzenazo III, (b) AAS and Ion selective electrode (ISE), and (c) ISE and Arzenazo III.
